# Bortezomib-induced hyponatremia: tolvaptan therapy permits continuation of lenalidomide, bortezomib and dexamethasone therapy in relapsed myeloma

**DOI:** 10.1186/s40164-019-0128-y

**Published:** 2019-02-01

**Authors:** N. O’Connor-Byrne, S. Glavey, R. Tudor, P. Murphy, C. J. Thompson, J. Quinn

**Affiliations:** 1 Academic Department of Haematology, Coleman Byrne Unit, Beaumont Hospital/RCSI Medical School, Dublin 9, Ireland; 2 Academic Department of Endocrinology, Beaumont Hospital/RCSI Medical School, Dublin, Ireland

**Keywords:** Hyponatremia, Multiple myeloma, Bortezomib, Tolvaptan

## Abstract

The development of hyponatremia due to syndrome of inappropriate antidiuretic hormone secretion (SIADH) is well recognised in multiple myeloma (MM). SIADH, due to either MM or Bortezomib can be hazardous as severe hyponatremia may develop if large volumes of hypotonic intravenous fluid are used as an adjunct to chemotherapy. We report a case of Bortezomib-induced SIADH, in whom the use of tolvaptan, a vasopressin receptor-2 antagonist, permitted the continuation of triple combination anti-MM therapy with lenalidomide, Bortezomib and dexamethasone (RVD) in a female with aggressive disease, without the development of hyponatremia. Our patient had a rapid relapse, in which the use of Bortezomib as part of an RVD regimen was life-saving. The use of tolvaptan allowed continuation of therapy that is usually halted in other similarly reported cases. This case highlights the possible use of vaptans, which allows an aquaresis to occur by blocking the antidiuretic effects of vasopressin, as a treatment for Bortezomib-induced hyponatremia.

## Background

Syndrome of inappropriate antidiuretic hormone secretion (SIADH) in a multiple myeloma (MM) patient treated with Bortezomib has been well documented in previous case reports. The presence of SIADH is not only associated with increased mortality [1] but complicates therapy for MM, as intravenous fluids can induce symptomatic severe hyponatremia when used as an adjunct to chemotherapy. We report a case of bortezomib-induced SIADH, in whom the use of tolvaptan, a vasopressin receptor-2 antagonist, allowed the continuation of combination anti-MM therapy with lenalidomide (revlimid, R), bortezomib (velcade, V) and dexamethasone (RVD) without symptomatic hyponatremia.

### Case

A 67-year-old female multiple myeloma (MM) patient presented with an aggressive MM relapse after recent autologous stem cell transplantation (ASCT). She had been diagnosed with MM 6 years previously and had been first treated with cyclophosphamide (Cy), thalidomide and dexamethasone, followed by high-dose melphalan and ASCT. Her disease returned 5 years later, and after re-induction treatment had a second ASCT but unfortunately relapsed again 3 months later, as indicated by pancytopenia, circulating plasma cells in the peripheral blood, an infiltrate of 90% plasma cells in the bone marrow and serum kappa light chains > 1800 mg/dL. Given the aggressive nature of disease at relapse, she commenced treatment with bortezomib 1.3 mg/m^2^ subcutaneously, (days 1, 4, 8, 11) and 40 mg dexamethasone (D) orally once daily (days 1–4, 9–12) in a 21-day cycle, with a plan to add lenalidomide in later cycles when the pancytopenia had improved.

**Fig. 1:**
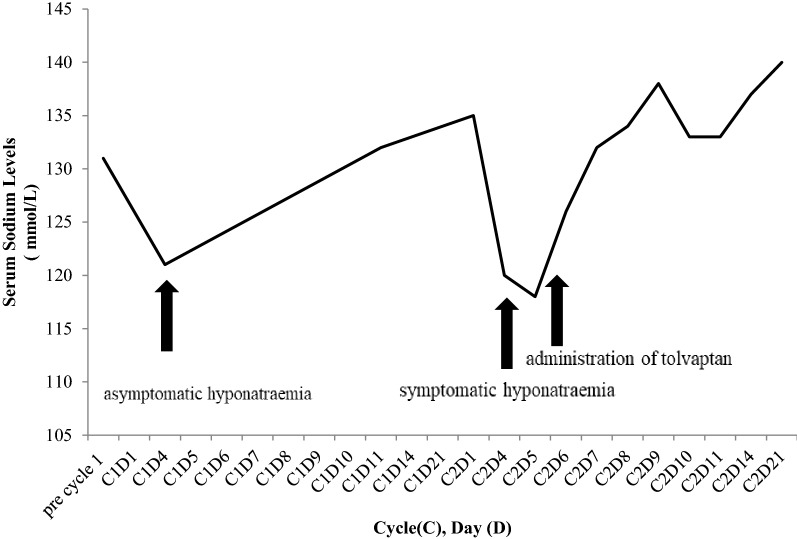
Hyponatraemia during anti-MM treatment and response to tolvaptan therapy

Although hyponatraemia occurred during the first cycle of treatment on day 8 (Fig. 1), there were no significant symptoms and the full cycle of treatment was completed with plasma sodium concentration returning to 135 mmol/L prior to commencement of cycle 2. However, on day 4 of cycle 2, the patient presented with nausea and abdominal pain. Clinical examination was unremarkable, however laboratory investigations revealed severe hyponatremia of 120 mmol/L (normal range 133–146 mmol/L). Urea was 4.2 mmol/L; urinary sodium was 70 mmol/L and urine osmolality was503 mOsm/kg. Thyroid function tests and serum cortisol levels were within normal ranges (TSH 1.13 mIU/L, normal range 0.38–5.33 mIU/L, free T4 9.2, normal range 7.0–13.0 pmol, morning cortisol 430 (normal range 185–624 nmol/L). Regular medications had been unchanged. Owing to her euvolemic volume status, hyponatremia, hypoosmolality, a urine osmolality > 100 mosmol/kg, urine sodium > 40 mmol/L and the timing of onset of hyponatremia, a diagnosis of bortezomib-induced SIADH was made. [2] Following endocrinology consultation a diagnosis of SIADH was made.

Fluid intake was initially restricted to 1500 mls daily and subsequently to 800 mL/24 h. Plasma sodium increased only marginally (118 to 121 mmol/L) over 2 days. Plasma sodium also responded poorly to two single intravenous boluses of 100 mL 3% saline over the following 24 h (122 to 126 mmol/L). The urgent need for chemotherapeutic treatment for disease relapse along with the requirement for adjunctive intravenous fluids, prompted escalation of therapy. Therefore, a trial of tolvaptan 7.5 mg orally once daily was commenced. There was a steady rise in plasma sodium concentration of 8 mmol over 24 h with resolution of the patient’s nausea (Table 1).

**Table 1:** Tolvaptan challenge—response of plasma sodium concentration to tolvaptan therapy

Time	09.00	12.00	14.40	18.00	08.00 following day
Plasma Na + (mmol/L)	126	127	130	131	132

When normonatremia was established, bortezomib was given on cycle 2 day 8, concurrent with tolvaptan when the serum sodium was then 134 mmol/L. Daily tolvaptan was continued, and the rest of the cycle proceeded without further episodes of hyponatremia (Fig. 1). The pancytopenia recovered fully and lenalidomide was commenced at a dose of 25 mg once daily as planned with cycle 3. The patient proceeded with bortezomib, lenalidomide, dexamethasone (RVD) along with tolvaptan, without hyponatremia. Tolvaptan was gradually tapered in the following months, owing to the distressing symptom of excessive thirst and stable normonatremia. Initially tolvaptan was tapered to alternate dosing of 7.5 mg once daily, 3 weeks after first being commenced. Seven weeks later, it was tapered to 3.75 mg, every 4 days. Five months after the initial episode, tolvaptan was discontinued entirely. The patient remains in remission nearly 24 months after starting RVD, which she still receives, without hyponatremia.

## Discussion

Bortezomib is a highly effective anti-MM agent. Hyponatremia due to SIADH is a known adverse effect of bortezomib, having been demonstrated in clinical trials [3, 4]. Our patient presented with severe symptomatic hyponatremia due to bortezomib-induced SIADH. Given the aggressive MM relapse, it was essential to reverse the hyponatremia and continue therapy with bortezomib. This prompted the use of tolvaptan to block the antidiuretic effects of the SIADH. This allowed the safe reversal of hyponatremia, symptomatic improvement and ultimately the continuation of effective RVD chemotherapy, without the redevelopment of severe symptomatic hyponatremia. In most cases of bortezomib-induced SIADH in the literature, hyponatremia has led to discontinuation of the bortezomib. In the only previously reported case where tolvaptan was used to treat SIADH during to bortezomib treatment, to the best of our knowledge, bortezomib therapy was not restarted at the time of reporting [5]. Of note, although lenalidomide-based anti-MM therapy may lead to hyponatremia [6], the introduction of lenalidomide to our patients’ regimen did not lead to fluctuations in the plasma sodium concentration.

The mechanism underlying bortezomib-induced SIADH is not fully understood but may mirrror the mechanisms the are implicated in the development of SIADH encountered with other commonly employed anti-cancer drugs such as cisplatin, cyclophosphamide, melphalan, vincristine and vinblastine [7]. For example, vincristine may cause SIADH by a direct toxic effect on the neurohypophysis and hypothalamic system, which alters the normal osmoreceptor control of ADH secretion [8] whereas high dose IV cyclophosphamide may cause SIADH by increasing ADH release and potentiating the effect of ADH [9]. Tolvaptan is a specific V2 receptor antagonist, which competitively inhibits vasopressin binding to the V2 receptors in the collecting tubules of the kidney. This allows an increase in free water clearance in SIADH, leading to a steady increase in the plasma sodium concentration [10].

The safety and efficacy of tolvaptan in the treatment of hyponatremia due to SIADH in cancer has been well documented, both in case series and in the setting of a double-blind, randomized, placebo-controlled clinical trial [11]. Its effects can last for up to 24 h, hence its initial daily dosing schedule [12]. In our case, tolvaptan was tapered because of the patient’s stable normonatremia on daily dosing for 3 weeks and the persistent discomforting symptoms of thirst. Given that the sodium remained normal for some weeks on alternate day dosing, it was tapered after approximately 2 months to 3.75 mg orally every 4 days and eventually discontinued, 5 months after the initial episode of hyponatremia.

Ideally, tolvaptan should be continued until the underlying disease is treated or and hyponatremia is no longer a clinical problem [13]. Whilst there are no clear guidelines on how tolvaptan should be discontinued [14], in the US, clinicians are required to limit its use to no more than 30 days due to the potential for liver toxicity with chronic use [12]. In cases of chronic hyponatraemia however, longer periods of therapy may be indicated on a case by case basis.

In conclusion, our case highlights the potential role of tolvaptan in the treatment of Bortezomib-induced SIADH in MM and importantly, provides a template for how effective RVD therapy can be delivered in this setting.

## Abbreviations

ASCT: autologous stem cell transplant
Cy: cyclophosphamide
CyBorD: cyclophosphamide, Bortezomib and dexamethasone
D: dexamethasone
MM: multiple myeloma
SIADH: syndrome of inappropriate antidiuretic hormone secretion
